# Obesity, chronic disease, age, and in-hospital mortality in patients with covid-19: analysis of ISARIC clinical characterisation protocol UK cohort

**DOI:** 10.1186/s12879-021-06466-0

**Published:** 2021-07-31

**Authors:** Thomas Yates, Francesco Zaccardi, Nazrul Islam, Cameron Razieh, Clare L. Gillies, Claire A. Lawson, Yogini Chudasama, Alex Rowlands, Melanie J. Davies, Annemarie B. Docherty, Peter J. M. Openshaw, J. Kenneth Baillie, Malcolm G. Semple, Kamlesh Khunti

**Affiliations:** 1 Diabetes Research Centre, Leicester General Hospital, University of Leicester, Leicester, LE5 4PW UK; 2 National Institute for Health Research (NIHR) Leicester Biomedical Research Centre (BRC), Leicester General Hospital, Leicester, LE5 4PW UK; 3 Leicester Real World Evidence Unit, Diabetes Research Centre, University of Leicester, Leicester, UK; 4 Clinical Trial Service Unit and Epidemiological Studies Unit (CTSU), Nuffield Department of Population Health, University of Oxford, Oxford, UK; 5 Medical Research Council Epidemiology Unit, University of Cambridge, Cambridge, UK; 6 Centre for Medical Informatics, Usher Institute, University of Edinburgh, Edinburgh, UK; 7 Intensive Care Unit, Royal Infirmary Edinburgh, Edinburgh, UK; 8 National Heart and Lung Institute, Imperial College London, London, UK; 9 Roslin Institute, University of Edinburgh, Edinburgh, UK; 10 NIHR Health Protection Research Unit in Emerging and Zoonotic Infections and Institute of Translational Medicine, Faculty of Health and Life Sciences, University of Liverpool, Liverpool, UK; 11 Respiratory Medicine, Alder Hey Children’s Hospital, Institute in The Park, University of Liverpool, Alder Hey Children’s Hospital, L12 2AP, Liverpool, UK; 12 NIHR Applied Research Collaboration – East Midlands (ARC-EM), Leicester General Hospital, Leicester, UK

**Keywords:** Obesity, Chronic disease, Ageing, COVID-19

## Abstract

**Background:**

Although age, obesity and pre-existing chronic diseases are established risk factors for COVID-19 outcomes, their interactions have not been well researched.

**Methods:**

We used data from the Clinical Characterisation Protocol UK (CCP-UK) for Severe Emerging Infection developed by the International Severe Acute Respiratory and emerging Infections Consortium (ISARIC). Patients admitted to hospital with COVID-19 from 6th February to 12th October 2020 were included where there was a coded outcome following hospital admission. Obesity was determined by an assessment from a clinician and chronic disease by medical records. Chronic diseases included: chronic cardiac disease, hypertension, chronic kidney disease, chronic pulmonary disease, diabetes and cancer. Mutually exclusive categories of obesity, with or without chronic disease, were created. Associations with in-hospital mortality were examined across sex and age categories.

**Results:**

The analysis included 27,624 women with 6407 (23.2%) in-hospital deaths and 35,065 men with 10,001 (28.5%) in-hospital deaths. The prevalence of chronic disease in women and men was 66.3 and 68.5%, respectively, while that of obesity was 12.9 and 11.1%, respectively. Association of obesity and chronic disease status varied by age (*p* < 0.001). Under 50 years of age, obesity and chronic disease were associated with in-hospital mortality within 28 days of admission in a dose-response manner, such that patients with both obesity and chronic disease had the highest risk with a hazard ratio (HR) of in-hospital mortality of 2.99 (95% CI: 2.12, 4.21) in men and 2.16 (1.42, 3.26) in women compared to patients without obesity or chronic disease. Between the ages of 50–69 years, obesity and chronic disease remained associated with in-hospital COVID-19 mortality, but survival in those with obesity was similar to those with and without prevalent chronic disease. Beyond the age of 70 years in men and 80 years in women there was no meaningful difference between those with and without obesity and/or chronic disease.

**Conclusion:**

Obesity and chronic disease are important risk factors for in-hospital mortality in younger age groups, with the combination of chronic disease and obesity being particularly important in those under 50 years of age. These findings have implications for targeted public health interventions, vaccination strategies and in-hospital clinical decision making.

**Supplementary Information:**

The online version contains supplementary material available at 10.1186/s12879-021-06466-0.

## Introduction

The severe acute respiratory syndrome coronavirus 2 (SARS-CoV-2), which causes coronavirus disease-2019 (COVID-19), is devastating global economies and putting unprecedented strain on health care services. The mortality and economic burden caused by the virus have precipitated an unparalleled global research response [1], including efforts to identify people at greatest risk of developing severe illness or mortality. Age, sex, chronic disease and obesity have emerged as key risk factors within large population level cohorts [2, 3]. Overweight and obesity have been suggested to increase the risk of severe disease or mortality by a factor of two [4], with diabetes, hypertension, coronary heart disease, chronic kidney disease, respiratory disease, and cancer all being shown to be associated with the risk of adverse COVID-19 outcomes [5–8]. However, whilst these factors have been extensively investigated individually, their interactions are less well understood. It is unknown, for example, whether obesity remains a strong risk factor in those without chronic disease, or whether the combination of obesity with chronic disease increases the risk, given their common coexistence. Whilst studies investigating the associations of obesity and chronic disease with COVID-19 outcomes have been adjusted for age, sex or both, adjustment can render invisible heterogeneity in associations across strata of interest which can mask important differences, including within COVID-19 research [9]. Early preliminary research in small cohorts has started to demonstrate the presence of heterogeneity for age, with obesity found to be more prevalent or a stronger risk factor for COVID-19 outcomes in younger compared to older populations [10–14], suggesting that younger people with obesity may be a priority group for public health testing and preventative strategies. However, not all research has supported these findings [15] and it is unknown whether age modifies associations between obesity and COVID-19 mortality equally in men and women or how obesity combines with associations of chronic disease. Examining how age modifies associations of combinations of common risk factors with COVID-19 outcomes will allow for more nuance and personalisation in risk communication, public health strategies and clinical decision making.

We use the ISARIC CCP-UK database to investigate associations between obesity, chronic disease and their combination with in-hospital death in patients admitted with COVID-19 and whether these associations are modified by age in men and women.

## Methods

### Study population

This study uses data from the Clinical Characterisation Protocol UK (CCP-UK) for Severe Emerging Infection developed by the International Severe Acute Respiratory and emerging Infections Consortium (ISARIC) and the World Health Organisation in response to the A/H1N1pdm2009 influenza pandemic [16]. ISARIC CCP-UK was reactivated in the UK on 17th January 2020 as the scale of the COVID-19 pandemic started to emerge. Data was collected from 260 hospitals in England, Scotland, and Wales. The protocol, amendment history, case report form, information leaflets, consent forms, and detail of the Independent Data and Material Access Committee (IDAMAC) are available at https://isaric4c.net. The study was approved by the South Central - Oxford C Research Ethics Committee in England (Ref: 13/SC/0149), and by the Scotland Research Ethics Committee (Ref: 20/SS/0028). For this study, we included those with a coding of “Proven or high likelihood of infection with a pathogen of Public Health Interest” reflecting that a preparedness protocol cannot assume a diagnostic test will be available for an emergent pathogen. Site training also emphasised importance of only recruiting proven cases of COVID-19. Additional inclusion criteria were: a hospital admittance date from the start of the COVID-19 pandemic in the UK (6th February 2020) with a complete follow-up coding indicating discharge, in-hospital death, or remaining in hospital. We excluded patients coded as having had a solid organ transplant or people with rare diseases and inborn errors of metabolism that significantly increase the risk of infections (such as severe combined immunodeficiency (SCID) or homozygous sickle cell). Data were available up until 12th October 2020. Of the 76,327 individuals within this dataset, 62,689 (82%) had a valid censoring date with complete data for age, sex, obesity and chronic disease (diabetes, chronic heart disease, hypertension, chronic kidney disease, chronic pulmonary disease or cancer) and were included in this analysis.

### Data collection

Data collection was undertaken by research nurses, administrators and medical students. Detailed admission data were collected on day 1 with follow-up data on disease progression collected on day 3, 6, and 9, and on discharge, death or continued hospitalisation beyond 28 days. Discharge was further coded as discharged alive, discharged to palliative care, or discharged to another facility.

Obesity was coded as yes/no on assessment from the attending clinician. Clinical assessment was based on objective measurement of obesity, such as by calculation of the body mass index (≥ BMI of 30 kg/m^2^), measurement of abdominal girth or on clinical judgment.

Chronic disease was based on clinician diagnosed status. In this study, we included diseases that have been consistently associated with COVID-19 outcomes [5–8]: chronic cardiac disease (coronary artery disease, heart failure, congenital heart disease, cardiomyopathy, rheumatic heart disease), hypertension, chronic kidney disease (diagnosed chronic kidney disease or estimated glomerular filtration rate < 60 mL/min/1.73m^2^), chronic pulmonary disease (chronic obstructive pulmonary disease [chronic bronchitis, emphysema], cystic fibrosis, bronchiectasis, interstitial lung disease, pre-existing requirement for long term oxygen therapy), diabetes (type 1 or 2), and malignant neoplasm (current solid organ or haematological malignancy).

### Outcome

The main outcome was in-hospital mortality coded as “death” for the patient outcome following hospital admittance. In order to avoid those with prolonged hospital stay biasing results, outcomes were censored at 28-days.

### Statistical analysis

The main analysis used a stratified rather than an adjusted model to investigate heterogeneity across strata. Data were stratified by age (< 50 years, 50–59 years, 60–69 years, 70–79 years, ≥80 years) and sex. Obesity and chronic disease were categorised into four mutually exclusive categories of interest: 1) non-obese without chronic disease, 2) obese without chronic disease, 3) non-obese with chronic disease, 4) obese with chronic disease. The Kaplan–Meier survival function was used to plot survival and generate survival tables. The log-rank test was used to determine overall differences in survival rates between obesity and chronic disease categories. Cox proportional hazards models were further used to quantify the relative hazard by age and sex strata for each combination of obesity and chronic disease compared to patients with no obesity and no chronic disease. In order to formally test whether age modified associations, an interaction test for age category by obesity/chronic disease category was also analysed, separately in men and women.

Two sensitivity analyses were conducted. The first considered the potential for discharge to palliative care biasing results, particularly in older individuals, by repeating analysis for a composite outcome of in-hospital mortality or discharge to palliative care. The second sensitivity analysis included obesity and each chronic disease separately to confirm whether the overall pattern of association observed in the main analysis was consistent for each individual disease and obesity. All analysis was conducted in SPSS (v26). *P* < 0.05 was used to denote significance for main effects and interactions. Data are reported as mean (95% CI), unless reported otherwise.

## Results

Table 1 shows the age, chronic disease and obesity characteristics of the included cohort. The analysis included 27,624 women and 35,065 men, with 6407 (23.2%) and 10,001 (28.5%) in-hospital deaths, respectively. The median number of days until discharge or death was between 8 and 9 days for both men and women. The proportion of patients with at least one chronic disease was 66.3 and 68.5% in women and men, respectively, while the prevalence of obesity was 12.9 and 11.1%, respectively. Rates of chronic disease and obesity by age group are shown in Supplementary Table S1, with rates of chronic disease highest in those over 80 years of age (83.6%) and rates of obesity highest in those 50–59 years of age (20.5%) and lowest in those over 80 years of age (4.7%).

**Table 1:** Participant characteristics stratified by sex

***Categorical Variables***	**Men (*n*** **= 35,065)**			**Women (27,624)**		
	**Number**	**Column %**		**Number**	**Column %**	
Age Category						
*< 50 years*	5398	15.4		4586	16.6	
*50–59 years*	4408	12.6		2745	9.9	
*60–69 years*	5789	16.5		3587	13.0	
*70–79 years*	8187	23.3		5581	20.2	
*80 years*	11,283	32.2		11,125	40.3	
Hypertension	9756	46.4		8234	46.3	
Chronic Heart Disease	11,491	33.2		7651	28.1	
Diabetes	8379	24.8		5364	20.1	
Chronic pulmonary disease	5985	17.3		4449	16.3	
Chronic Kidney Disease	5553	16.0		4583	16.8	
Cancer	4197	12.1		3161	11.6	
Any chronic disease	24,007	68.5		18,316	66.3	
Obesity	3901	11.1		3561	12.9	
Mutually exclusive categories of obesity and chronic disease						
*Non obese, no chronic disease*	10,103	28.8		8259	29.9	
*Obese, no chronic disease*	955	2.7		1049	3.8	
*Non-obese, with chronic disease*	21,061	60.1		15,804	57.2	
*Obese with chronic disease*	2946	8.4		2512	9.1	
In-hospital mortality within 28 days	10,001	28.5		6407	23.2	
Discharged within 28 days	20,388	58.1		17,625	63.8	
Remain in hospital after 28 days	4676	13.3		3592	13.0	
***Continuous Variables***	**Median**	**25th percentile**	**75th percentile**	**Median**	**25th percentile**	**75th percentile**
Days in hospital until death	9	4	16	8	4	16
Days in hospital until discharge	8	4	16	8	4	16

**Fig. 1:**
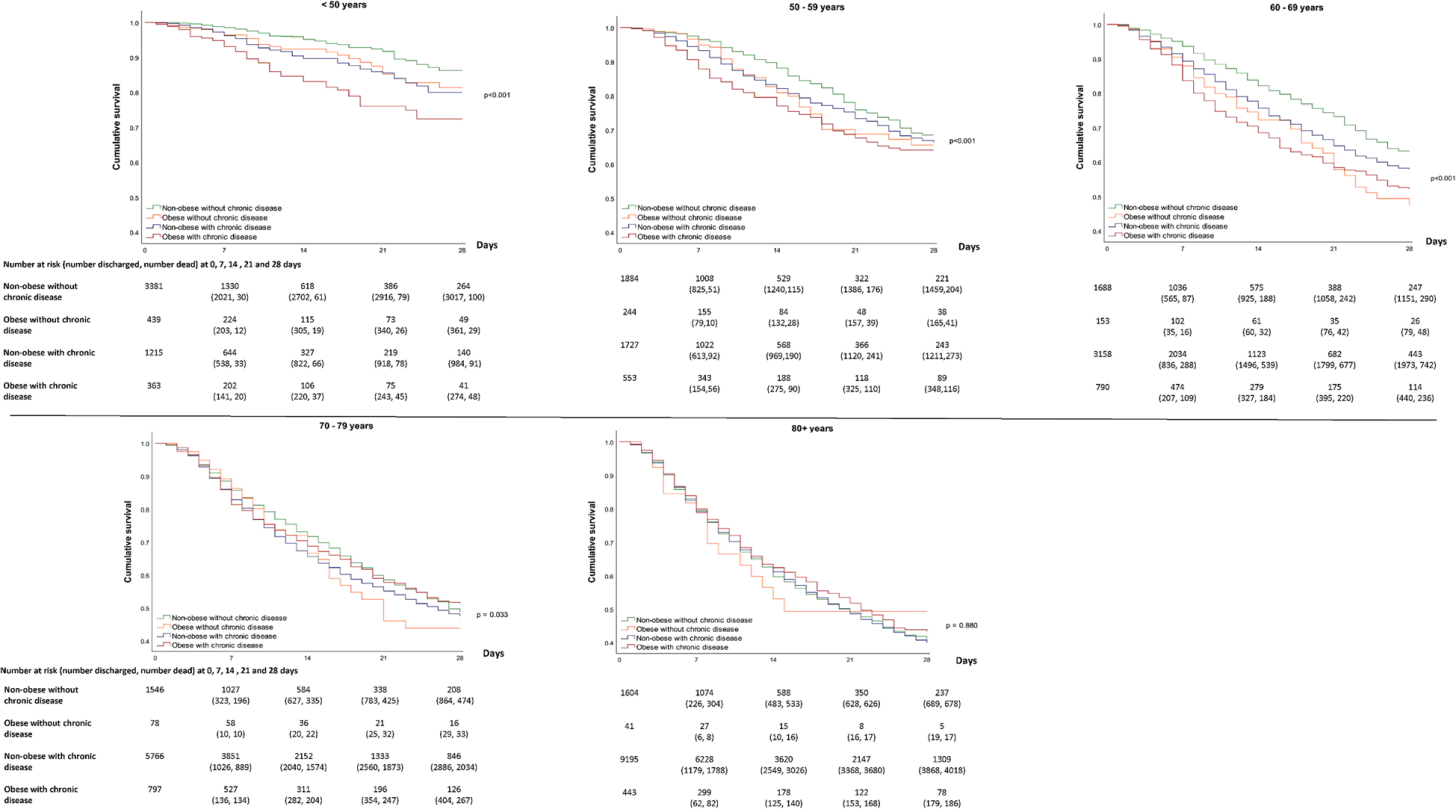
28-day survival in men across mutually exclusive categories of obesity and chronic disease by age

**Fig. 2:**
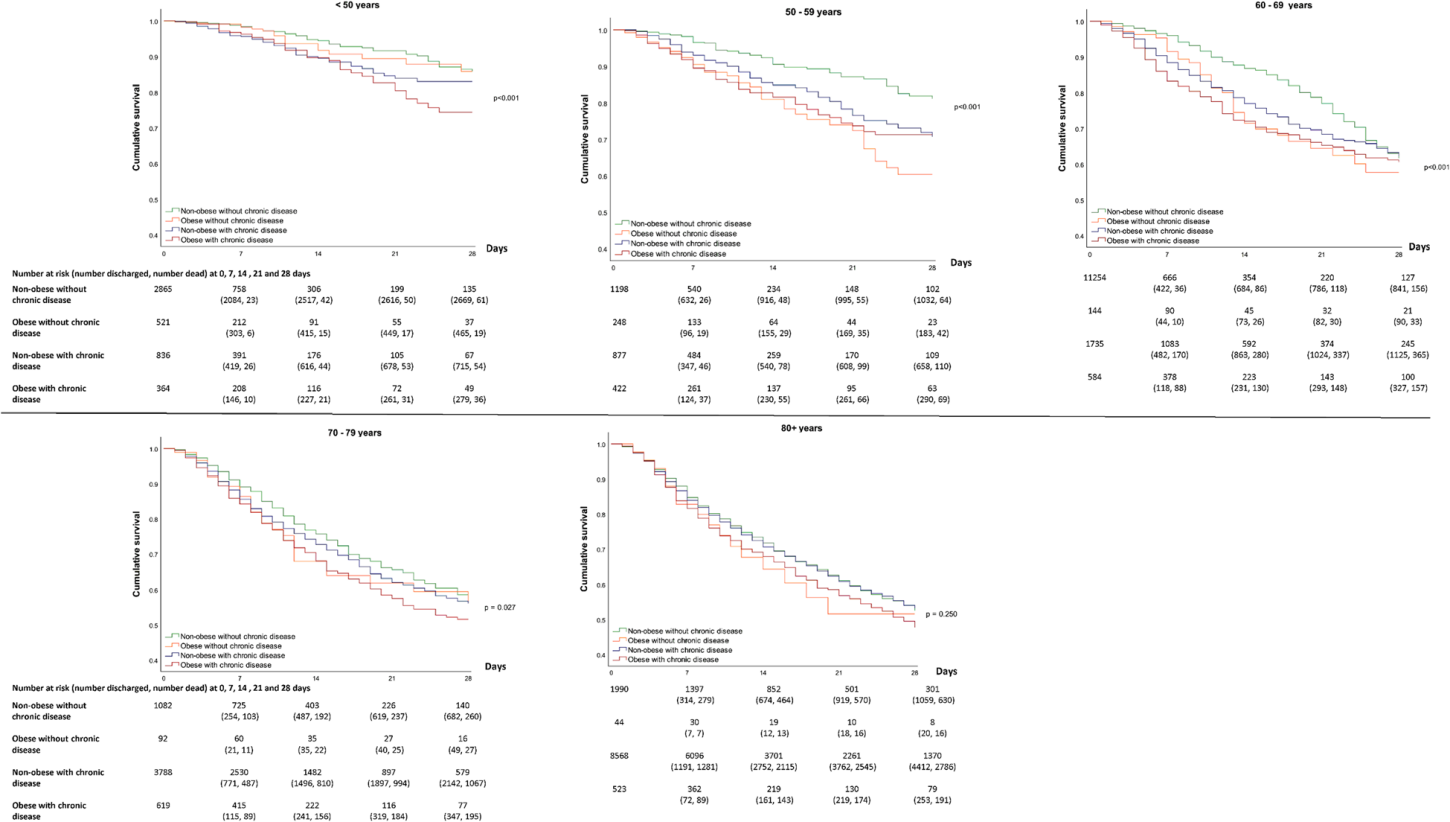
28-day survival in women across mutually exclusive categories of obesity and chronic disease by age

**Fig. 3:**
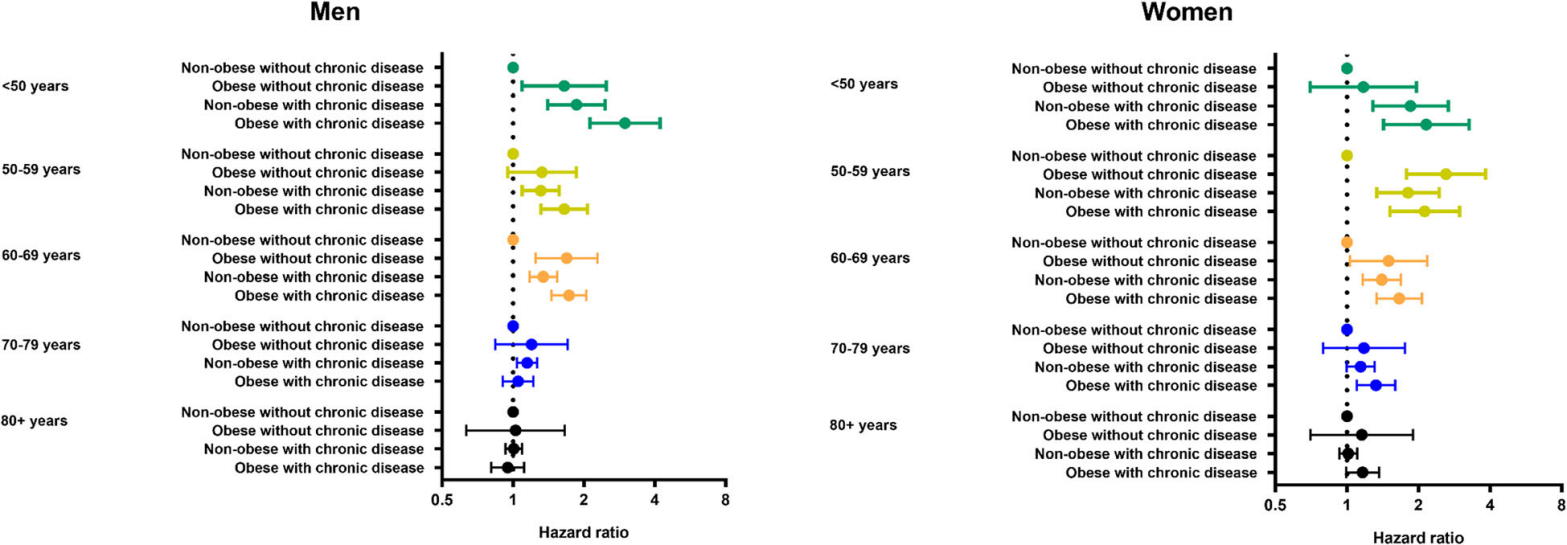
Risk of in-hospital mortality in men and women across mutually exclusive categories of obesity and chronic disease by age

Figure 1 for men and Fig. 2 for women show the 28-day survival across obesity, chronic disease and age categories, with Fig. 3 showing the accompanying hazard ratios (data shown in Supplementary Table S2). Associations between obesity and chronic disease categories with in-hospital mortality varied by age in men and women (*P* < 0.001 for interaction).

For patients under 50 years of age, obesity and chronic diseases were associated with in-hospital mortality in a dose-response manner, such that those with both obesity and chronic disease had the lowest survival (Figs. 1 and 2), with a HR of 2.99 (95% CI: 2.12, 4.21) in men and 2.16 (1.42, 3.26) in women compared to patients without obesity or chronic diseases (Fig. 3). In the age categories of 50–59 and 60–69 years, obesity and chronic disease remained associated with in-hospital COVID-19 mortality, but survival in those with obesity tended to be similar in those with and without coexisting chronic disease (Fig. 3). For example, in women between 50 and 59 years, compared to those without obesity or chronic disease, the HR of in-hospital mortality in those with obesity without chronic disease was 2.61 (1.78, 3.82) while the HR in those with obesity and chronic disease was 2.12 (1.52, 2.93). Between 70 and 79 years, there was no clear pattern of association between obesity and chronic disease in men, whereas risk in those with obesity and chronic disease remained moderately elevated in women (HR = 1.33; 1.10, 1.60) (Fig. 3). Beyond 80 years of age, survival was low across all categories of obesity and chronic disease status (Figs. 1 and 2) with no meaningful difference in survival between those without obesity and chronic disease compared to those with obesity and/or chronic disease in men and women.

The strength and pattern of association was unchanged when those discharged to palliative care (men = 437, women = 459) were included in a composite outcome with mortality (Supplementary Figure S1). The pattern of association with in-hospital mortality was also consistent across categories of age when obesity and each included chronic disease were analysed individually (Supplementary Figure S2), with associations weakest in older adults.

## Discussion

In patients admitted to hospital with COVID-19, mutually exclusive categories of obesity and chronic disease are consistently associated with in-hospital mortality in younger adults but not in those 70 years of age or over for men or 80 years and over for women. In men and women under 50 years of age, obesity and the coexistence of chronic diseases combined to increase the risk of in-hospital mortality by between 2 to 3 times compared to those without obesity or chronic diseases. Between the ages of 50 to 69 years, obesity and chronic diseases remained associated with a higher risk of in-hospital mortality in men and women, but their combination did not appear to further reduce survival beyond having obesity only. In those aged 70 years or over in men and 80 or over years in women, survival was low across all combinations of obesity and chronic disease, but was not meaningfully further lowered in those with obesity and/or chronic disease.

Whilst obesity and chronic disease have been established as risk factors for SARS-CoV-2 infection, severe disease and mortality [2–8], there has been less focus on establishing how these risk factors combine in men and women and whether the pattern of association is consistent across different age groups. This study expands preliminary findings from smaller cohorts suggesting obesity may be a particularly important risk factor in younger ages. Among 7606 patients in the United States, associations of BMI with in-hospital mortality and mechanical ventilation were strongest in those under 50 years and largely attenuated in those over 70 years [14], which was consistent with an early study from a New York hospital system [10]. Another study from the USA found that in 6916 persons testing positive for COVID-19, obesity was more strongly associated with mortality in younger age men [12], while a study from Spain involving 1105 persons hospitalised with COVID-19 also reported that obesity was more strongly associated with admission in younger adults [13]. However, a study involving 5795 persons admitted to hospitals in Paris with COVID-19 suggested obesity was similarly associated with mortality across age groups [15], although uncertainty around the estimates of risk were high due to a low number of events when stratified by age. The present study suggests that, with cases of COVID-19 that are severe enough to warrant admission to hospital, the coexistence of obesity and chronic disease substantially reduces survival in those under 50 years of age, with obesity remaining a risk factor with or without the coexistence of chronic disease up until the age of 70 years. However, beyond this age, obesity with or without chronic disease may not meaningfully affect the already low levels of survival associated with older age, particularly in men. These findings broadly support the vaccination strategy within the United Kingdom [17], the first country to approve the roll-out of a COVID-19 vaccine. The strategy sets out health care workers and older adults (with priority set in descending age order until ≥65 years) as the first priority, followed by those over 16 years of age with an underlying health condition, including morbid obesity and the chronic diseases included in this paper.

Strengths of this analysis include the large multisite sample with data collected from trained staff according to standard operating procedures. In the United Kingdom (UK), there were a total of 151,641 hospital admissions with COVID-19 up until October 12th (https://coronavirus.data.gov.uk/). Therefore, our cohort represents 41% of all UK hospital admissions in the period covered by this study. This large sample size allowed for stratification by age, sex and mutually exclusive categories of obesity and chronic diseases, allowing survival estimates for each strata rather than relying on adjusted estimates which can mask important differences between groups. However, there are some limitations. Namely, the large sample size was only made possible by relying on clinical records: for example, obesity was defined by a clinician assessment. Therefore, the prevalence of obesity appears an under-estimate compared to levels that would have been expected based on population level estimates [18]. It is likely the coding of obesity in this study therefore reflects more extreme phenotypes of obesity likely to prompt a clinical coding. Other factors like age may also influence whether patients were assessed for obesity, which could act to bias the reported associations. Nevertheless, in order to inform clinical care, analysed risk factors need to reflect data that is readily available to treating clinical staff through clinical records. Therefore, the coding of obesity in this study may have real world utility as it corresponds to data coded within routine clinical care. Another potential limitation is that this study is specific to in-hospital mortality and does not include COVID-19 mortality events in the community. Post-acute COVID-19 discharge from hospital of patients into palliative care is increasingly recognised with ‘long-COVID-19’ [19], which may have acted to bias findings through longer-term hospital admissions followed by a high mortality risk once discharged. Nevertheless, associations were unchanged when patients discharged into palliative care were included within the outcome as a composite with mortality.

In conclusion, this study suggests obesity and chronic diseases act as important risk factors for lower in-hospital survival in younger age groups, with the combination of chronic disease and obesity being a particularly important risk factor in patients under 50 years of age. Associations appeared to be attenuated with age, such that obesity and chronic diseases had little impact on survival in men beyond 70 years and women beyond 80 years. These findings suggests that younger adults with obesity and pre-existing chronic disease may need to be targeted by public health interventions to reduce the risk of transmission or to engage with vaccination programmes. However, obesity and chronic disease may be less important or informative for clinical decision making in the treatment of COVID-19 for older adults, where other factors may be more relevant.

## Supplementary Information

**Additional file 1: Supplementary Table S1.** Rates of obesity and chronic disease by age group. **Supplementary Table S2.** Hazard ratio estimates used to draw Fig. 3. **Supplementary Figure S1.** Hazard ratios for each strata when those discharged into palliative care were included with in-hospital mortality as a composite outcome. **Supplementary Figure S2.** Hazard ratios for each disease and obesity analysed individually**.**

## Data Availability

The data that support the findings of this study are available from International Severe Acute Respiratory and emerging Infections Consortium (ISARIC) but restrictions apply to the availability of these data, which were used under license for the current study, and so are not publicly available. Data are however available from the authors upon reasonable request and with permission of ISARIC4C. If someone wishes to request the data from this study, please contact the ISARIC4C consortium (https://isaric4c.net/) for access enquiries.
