# Supplementary material for: Obesity, chronic disease, age, and in-hospital mortality in patients with covid-19: analysis of ISARIC clinical characterisation protocol UK cohort
Source: BMC Infect Dis. 2021 Jul 31;21:717. doi: 10.1186/s12879-021-06466-0 (PMC8324452; doi:10.1186/s12879-021-06466-0)
Supplement: Supplementary file 1 — Additional file 1: Supplementary Table S1. Rates of obesity and chronic disease by age group. Supplementary Table S2. Hazard ratio estimates used to draw Fig. 3. Supplementary Figure S1. Hazard ratios for each strata when those discharged into palliative care were included with in-hospital mortality as a composite outcome. Supplementary Figure S2. Hazard ratios for each disease and obesity analysed individually. [file 12879_2021_6466_MOESM1_ESM.docx]

**OBESITY, CHRONIC DISEASE, AGE, AND IN-HOSPITAL MORTALITY IN PATIENTS WITH COVID-19: ANALYSIS OF ISARIC Clinical Characterisation Protocol UK cohort**

Thomas Yates, PhD^1, 2^ [ty20@leicester.ac.uk](mailto:ty20@leicester.ac.uk)

Francesco Zaccardi, PhD^1, 3^ [fz43@leicester.ac.uk](mailto:fz43@leicester.ac.uk)

Nazrul Islam, PhD^4, 5^ [nazrul.Islam@ndph.ox.ac.uk](mailto:nazrul.Islam@ndph.ox.ac.uk)

Cameron Razieh, MSc^1, 2^ [cr288@leicester.ac.uk](mailto:cr288@leicester.ac.uk)

Clare L Gillies, PhD^1, 3^ [clg13@leicester.ac.uk](mailto:clg13@leicester.ac.uk)

Claire A. Lawson, PhD^1^ [cl417@leicester.ac.uk](mailto:cl417@leicester.ac.uk)

Yogini Chudasama, PhD^3^ [yc244@leicester.ac.uk](mailto:yc244@leicester.ac.uk)

Alex Rowlands, PhD^1, 2^ [alex.rowlands@leicester.ac.uk](mailto:alex.rowlands@leicester.ac.uk)

Melanie J Davies, MD^1, 2^ [melanie.davies@uhl-tr.nhs.uk](mailto:melanie.davies@uhl-tr.nhs.uk)

Annemarie B Docherty, PhD^6,7^ [annemarie.docherty@ed.ac.uk](mailto:annemarie.docherty@ed.ac.uk)

Peter JM Openshaw, PhD^8^ [p.openshaw@imperial.ac.uk](mailto:p.openshaw@imperial.ac.uk)

J Kenneth Baillie, PhD^9^ [j.k.baillie@ed.ac.uk](mailto:j.k.baillie@ed.ac.uk)

Malcolm G Semple, PhD^10,11^ [M.G.Semple@liverpool.ac.uk](mailto:M.G.Semple@liverpool.ac.uk)

ISARIC4C investigators*

Kamlesh Khunti, PhD^1, 3, 12^ [kk22@leicester.ac.uk](mailto:kk22@leicester.ac.uk)

1. Diabetes Research Centre, University of Leicester, Leicester General Hospital, Leicester, LE5 4PW, UK.
2. National Institute for Health Research (NIHR) Leicester Biomedical Research Centre (BRC), Leicester General Hospital, Leicester, LE5 4PW, UK.
3. Leicester Real World Evidence Unit, Diabetes Research Centre, University of Leicester, Leicester, UK.
4. Clinical Trial Service Unit and Epidemiological Studies Unit (CTSU), Nuffield Department of Population Health, University of Oxford, Oxford, UK.
5. Medical Research Council Epidemiology Unit, University of Cambridge, Cambridge, UK.
6. Centre for Medical Informatics, Usher Institute, University of Edinburgh, Edinburgh, UK
7. Intensive Care Unit, Royal Infirmary Edinburgh, Edinburgh, UK
8. National Heart and Lung Institute, Imperial College London, London, UK
9. Roslin Institute, University of Edinburgh, Edinburgh, UK
10. NIHR Health Protection Research Unit in Emerging and Zoonotic Infections and Institute of Translational Medicine, Faculty of Health and Life Sciences, University of Liverpool, Liverpool, UK
11. Respiratory Medicine, Alder Hey Children’s Hospital, Institute in The Park, University of Liverpool, Alder Hey Children’s Hospital, Liverpool L12 2AP, UK
12. NIHR Applied Research Collaboration – East Midlands (ARC-EM), Leicester General Hospital, Leicester, UK.

*ISARIC4C investigators listed in end material

***Corresponding author*:** Prof Tom Yates; Tel: 0116 258 6481; Fax: 0116 258 4499; Email: [ty20@le.ac.uk](mailto:ty20@le.ac.uk); Address: Diabetes Research Centre, University of Leicester, Leicester General Hospital, Gwendolen Road, Leicester, LE5 4PW.

**Supplementary Table S1: Rates of obesity and chronic disease by age group**

| Factor | | < 50 years | 50 – 59 years | 60 – 69 years | 70 – 79 years | 80+ years |
| --- | --- | --- | --- | --- | --- | --- |
| Obesity | No | 8297 (83.1) | 5686 (79.5) | 7705 (82.2) | 12182 (88.5) | 21357 (95.3) |
|  | Yes | 1687 (16.9) | 1467 (20.5) | 1671 (17.8) | 1586 (11.5) | 1051 (4.7) |
| Chronic disease | No | 7206 (72.2) | 3574 (50.0) | 3109 (33.2) | 2798 (20.3) | 3679 (16.4) |
|  | Yes | 2778 (27.8) | 3579 (50.0) | 6267 (66.8) | 10970 (79.7) | 18729 (83.6) |

Data as number (%)

**Supplementary Table S2: Hazard ratio estimates used to draw Figure 3**

**Men**

|  | < 50 years | | | 50 - 59 years | | | 60 - 69 years | | | 70 -79 years | | | ≥ 80 years | | |
| --- | --- | --- | --- | --- | --- | --- | --- | --- | --- | --- | --- | --- | --- | --- | --- |
|  | HR | Lower CI | Upper CI | HR | Lower CI | Upper CI | HR | Lower CI | Upper CI | HR | Lower CI | Upper CI | HR | Lower CI | Upper CI |
| Non-obese healthy | 1.00 | 1.00 | 1.00 | 1.00 | 1.00 | 1.00 | 1.00 | 1.00 | 1.00 | 1.00 | 1.00 | 1.00 | 1.00 | 1.00 | 1.00 |
| Obese healthy | 1.65 | 1.09 | 2.49 | 1.33 | 0.95 | 1.86 | 1.69 | 1.25 | 2.29 | 1.20 | 0.84 | 1.71 | 1.03 | 0.63 | 1.66 |
| Non-obese chronic disease | 1.86 | 1.40 | 2.46 | 1.31 | 1.09 | 1.57 | 1.34 | 1.17 | 1.54 | 1.15 | 1.04 | 1.27 | 1.01 | 0.93 | 1.09 |
| Obese chronic disease | 2.99 | 2.12 | 4.21 | 1.65 | 1.31 | 2.07 | 1.73 | 1.46 | 2.05 | 1.05 | 0.91 | 1.22 | 0.95 | 0.81 | 1.12 |

**Women**

|  | < 50 years | | | 50 - 59 years | | | 60 - 69 years | | | 70 -79 years | | | ≥ 80 years | | |
| --- | --- | --- | --- | --- | --- | --- | --- | --- | --- | --- | --- | --- | --- | --- | --- |
|  | HR | Lower CI | Upper CI | HR | Lower CI | Upper CI | HR | Lower CI | Upper CI | HR | Lower CI | Upper CI | HR | Lower CI | Upper CI |
| Non-obese healthy | 1.00 | 1.00 | 1.00 | 1.00 | 1.00 | 1.00 | 1.00 | 1.00 | 1.00 | 1.00 | 1.00 | 1.00 | 1.00 | 1.00 | 1.00 |
| Obese healthy | 1.17 | 0.70 | 1.96 | 2.61 | 1.78 | 3.83 | 1.50 | 1.03 | 2.18 | 1.18 | 0.79 | 1.75 | 1.16 | 0.70 | 1.90 |
| Non-obese chronic disease | 1.85 | 1.29 | 2.67 | 1.81 | 1.33 | 2.44 | 1.40 | 1.16 | 1.69 | 1.14 | 1.00 | 1.31 | 1.01 | 0.93 | 1.10 |
| Obese chronic disease | 2.16 | 1.42 | 3.26 | 2.12 | 1.52 | 2.97 | 1.66 | 1.33 | 2.07 | 1.33 | 1.10 | 1.60 | 1.16 | 0.99 | 1.37 |

**Supplementary Figure S1: Hazard ratios for each strata when those discharged into palliative care were included with in-hospital mortality as a composite outcome**


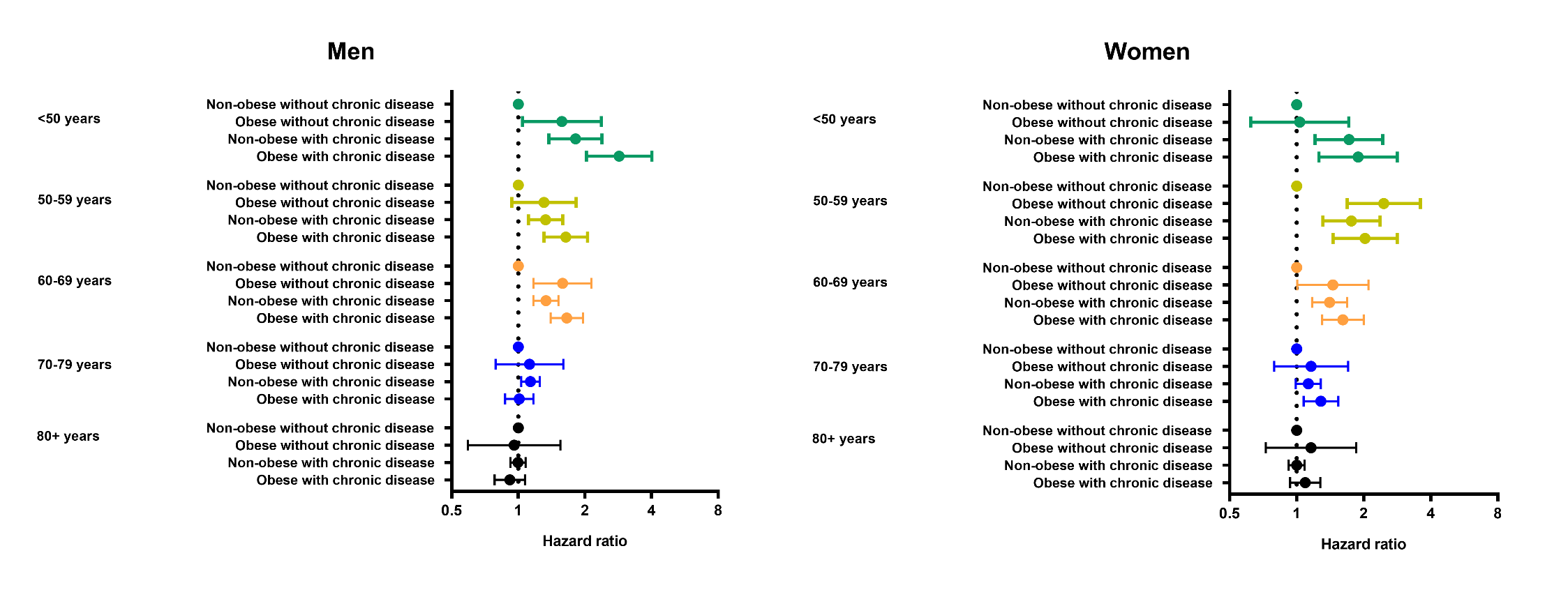


**Supplementary Figure S2: Hazard ratios for each disease and obesity analysed individually**

**
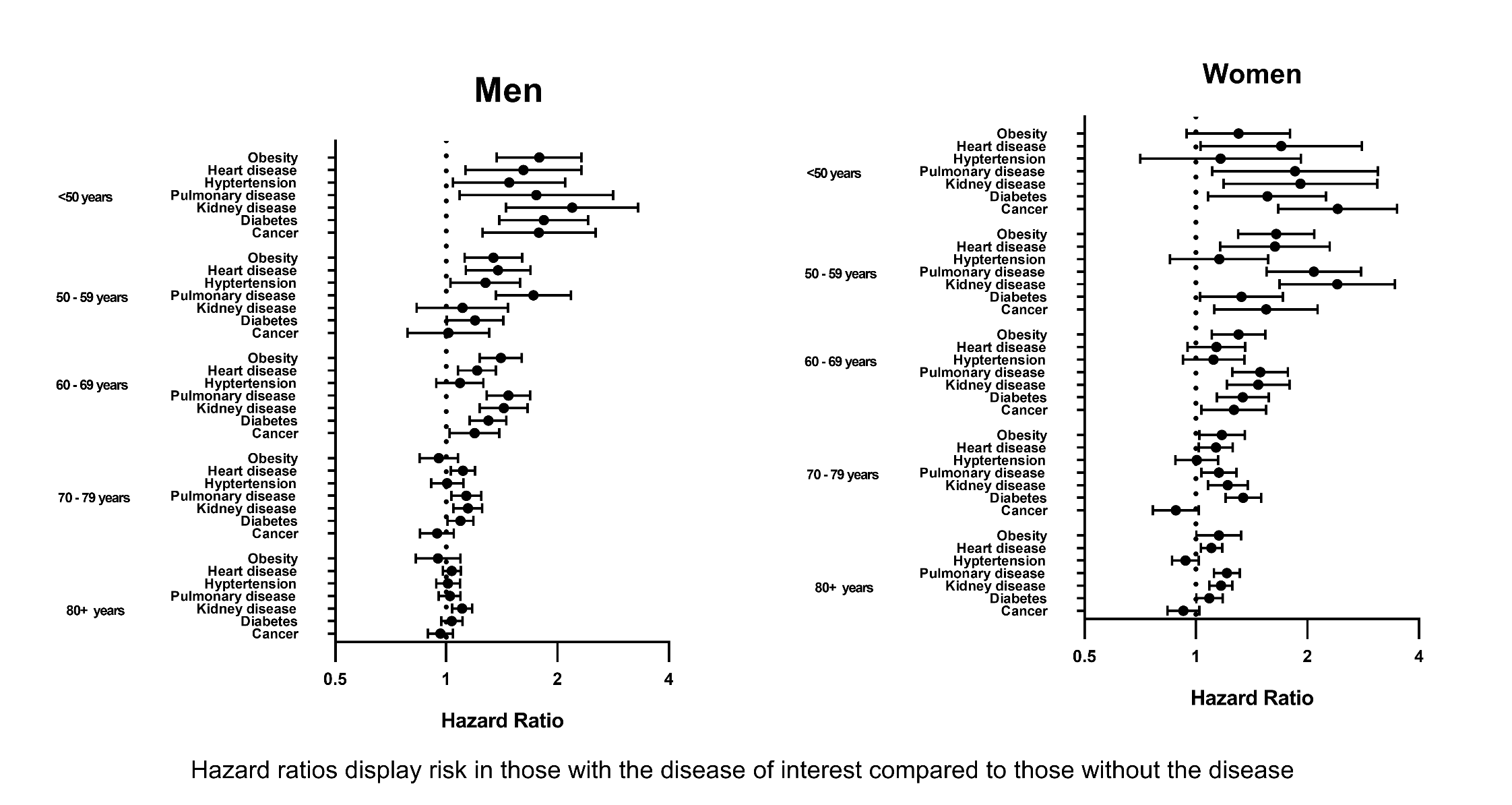
**
